# Knowledge, attitudes, and practices related to anthrax and animal care: A case-control study in Georgia

**DOI:** 10.1371/journal.pone.0224176

**Published:** 2019-10-18

**Authors:** Rita M. Traxler, Tsira Napetvaridze, Zviad Asanishvili, Marika Geleishvili, Ketevan Rukhadze, Giorgi Maghlakelidze, Mariam Broladze, Maka Kokhreidze, Edmond F. Maes, Debby Reynolds, Mo Salman, Sean V. Shadomy, Sangeeta Rao

**Affiliations:** 1 Centers for Disease Control and Prevention (CDC), Division of High-Consequence Pathogens and Pathology, National Center for Emerging and Zoonotic Infectious Diseases (NCEZID), Atlanta, Georgia, United States of America; 2 National Food Agency (NFA) of Ministry of Environmental Protection and Agriculture of Georgia (MEPA), Tbilisi, Georgia; 3 CDC, Division of Global Health Protection, Center for Global Health, Atlanta, Georgia, United States of America; 4 Department of Rural Development and Vocational Education (DRDVE) of Georgian Institute of Public Affairs (GIPA), Tbilisi, Georgia; 5 National Center for Disease Control and Prevention (NCDC), Tbilisi, Georgia; 6 Laboratory of the Ministry of Agriculture (LMA), Tbilisi, Georgia; 7 CDC, Global Immunization Division, Center for Global Health, Atlanta, Georgia, United States of America; 8 Department of Clinical Sciences, Colorado State University, Fort Collins, Colorado, United States of America; 9 CDC One Health Office, NCEZID, Atlanta, Georgia, United States of America; Spectrum Health, UNITED STATES

## Abstract

**Introduction:**

Anthrax is endemic in Georgia and recent outbreaks prompted a livestock-handler case-control study with a component to evaluate anthrax knowledge, attitudes, and practices (KAP) among livestock handlers or owners.

**Methods:**

Cases were handlers of livestock with confirmed animal anthrax from June 2013-May 2015. Handlers of four matched unaffected animals were selected as controls, two from the same village as the case animal (“village control”) and two from 3–10 km away (“area control”). Descriptive statistics were reported and conditional logistic regression was performed to estimate the magnitude of the association of cases with specific study KAP factors.

**Results:**

Cases were more likely male, had lower level college education, less animal care experience, and provided more animal care to their cattle. Cases had lower odds of burying a suddenly dead animal compared to all controls (Odds Ratio [OR]: 0.32, 95% Confidence interval [CI]:0.12, 0.88) and area controls (OR: 0.32, 95% CI: 0.11, 0.91). On an 8-point knowledge scale, cases having an animal with anthrax had a 1.31 times greater knowledge score compared to all controls (95% CI: 1.03, 1.67). Cases had higher odds of ever having human anthrax or knowing another person who had anthrax compared to all controls (OR: 4.56, 95% CI: 1.45, 14.30) and area controls (OR: 7.16, 95% CI: 1.52, 33.80).

**Discussion:**

Cases were more knowledgeable of anthrax and had better anthrax prevention practices, but these are likely a result of the case investigation and ring vaccination following the death of their animal.

**Conclusions:**

The findings reveal a low level of knowledge and practices related to anthrax control and prevention, and will guide educational material development to fill these gaps.

## Introduction

Anthrax is a zoonotic disease primarily affecting herbivorous mammals caused by *Bacillus anthracis* spores [1]. Spores can survive for decades in soil, and the disease is endemic throughout the world [2]. Despite its endemicity, livestock handlers in endemic areas often have limited anthrax knowledge [3, 4]. Livestock are important assets, which can encourage the practice of salvage slaughtering and butchering. Mebratu et al. (2015) found that despite 95% of livestock handlers being aware of the risk of anthrax transmission to humans, 12.5% would still eat and sell the meat from an animal anthrax case to limit economic losses with low regard for the public health considerations [5].

Following a human anthrax outbreak in Georgia in 2012, where anthrax is considered endemic [6, 7], an investigation was undertaken to identify the risk factors for human infection [8]. In a previous published investigation, Navdarashvili et al. (2016) found that livestock handling practices including slaughtering animals, disposing of dead animals, and contact with sick animals were independent predictors of human anthrax.

Based on the investigation findings, a case-control study was conducted to identify factors contributing to disease among livestock [9] as the primary aim of the overall objective of the investigation. The current study was a part of the investigation with an aim to further identify gaps in anthrax knowledge, attitudes, and practices (KAP) among livestock handlers, from which targeted educational materials will be developed. We hypothesized that the caretakers of anthrax-infected animals would have lower knowledge of anthrax prior to the occurrence of the disease when compared to the knowledge of healthy animal caretakers.

## Materials and methods

### Study sample

A matched case-control study was conducted that included a KAP component. Cases were defined as the owners or handlers/shepherds (hereafter ‘handler’ unless expressly delineated) of mammalian livestock species with sudden unexplained death or anthrax-consistent signs pre- or post-mortem, and confirmed by either *B*. *anthracis* isolation from the carcass or *B*. *anthracis* virulence gene detection by PCR. All cases with onset or death between 1 June 2013 and 31 May 2015 were included.

The handlers of four animals of the same species as the case’s animal were selected as controls; two were randomly selected from the same village (“village controls”) and two were selected within 3–10 km from the case village (“area controls”). Village controls were included to examine differences between handler activities and knowledge specific to the individual animal, since animals within a village are often pastured together during the day overseen by a single shepherd. Separate area controls were included to examine risk factors and exposures that could affect herd-level management practices to understand what some herds are doing differently that puts them at risk for the disease. These controls were outside the “intervention zone” that received anthrax control measures following the animal anthrax death [10], but were proximal to expect similar anthrax risk.

Inclusion criteria for controls were that their animals were age >3 months and were owned by the control during the one-month period prior to the disease onset (Period 1) or death of the matched case animal if disease onset was unknown (Date 1). Cases and controls were ≥18 years of age, agreed to participate, and gave verbal consent. The full case definition and design is described elsewhere [10]; only cattle handlers are included in this analysis due to small numbers of other animal species in the study. The CDC National Center for Emerging and Zoonotic Infectious Diseases Human Subjects Advisor determined the study was a non-research activity, and ethical review by the NFA Department of Law was not required.

### Data collection

A standard questionnaire (available from the senior author) was developed in Georgian and Azeri languages; some questionnaires were translated orally into Russian when it was the only common language between the interviewer and participant. NFA staff conducted the interviews, accompanied by interpreters, trainers, and observers. Retrospective study enrollment occurred for cases with Date 1 between 1 June and 30 October 2013; prospective enrollment of cases occurred from 31 October 2013-September 2015.

### Measures

A knowledge scale was developed by summing together the scores (0 = No or 1 = Yes) from eight questions regarding the handler’s knowledge of anthrax in animals and humans (e.g., Can people get anthrax from animals that have anthrax?). A practices scale was developed from 11 questions about what is done with a sick or dead animal using the same scoring (e.g., Do you slaughter an animal when it suddenly becomes sick?). A higher score indicates a greater overall knowledge of anthrax or using appropriate practices when handling an animal suspected to have anthrax. Both measures were evaluated using Cronbach’s alpha to test internal reliability [11]; standardized alpha for knowledge was 0.73, and 0.31 for the practice measure. Due to the low alpha for the practices measure, only knowledge was retained in analyses.

### Data analysis

Questionnaire data were double entered (to check for data-entry errors) into an Epi Info 7 database (CDC, Atlanta, GA, USA); text fields were translated into English for analysis. Analyses were performed in SAS 9.4 (SAS Institute, Cary, NC, USA). Means, standard deviations, and frequencies were calculated. Cases were compared to village controls, area controls, and all combined controls using conditional logistic regression. Odds ratios as a measure of magnitude of association were estimated from the conditional logistic regression. The level of the statistical significance was α = 0.05.

## Results

The study participants consisted of 30 cases and 60 each matched area controls and village controls. Overall, 57% were male and the average age was 53.3 years (Standard deviation [SD]: 14.4). Cases were more often males (75%), while controls were about 52% male. The most frequent educational attainment was completion of secondary school (48%) while 40% had additional schooling; the breakdown by cases and controls is in Table 1. Only 28% of cases had additional schooling beyond secondary school, compared to 44% among village controls and 42% of area controls, although this difference was not significantly associated with anthrax.

**Table 1:** Demographics of handlers of cattle with anthrax (‘Cases’) and handlers of matched uninfected cattle (‘Controls’) in Georgia, June 2013–May 2015.

Demographic variable	Cases (N = 30)	Village Controls (N = 60)	Area Controls (N = 60)
	N^ (%)	N^ (%)	N^ (%)
Mean age in years (SD)	52.6 (13.8)	51.1 (14.7)	56.0 (14.1)
Sex (Male)	21/28 (75)	31/59 (52.5)	30/57 (52.6)
Education	**(N = 29)**	**(N = 59)**	**(N = 60)**
Primary or some secondary	5 (17.2)	6 (10.2)	7 (11.7)
Completed secondary	16 (55.2)	27 (45.8)	28 (46.7)
Certificate or some college	5 (17.2)	12 (20.3)	10 (16.7)
Completed college	3 (10.3)	14 (23.7)	15 (25)
Time caring for animal in Period 1#	**(N = 30)**	**(N = 60)**	**(N = 58)**
All the time	15 (50)	17 (28.3)	16 (27.6)
Morning and evening	13 (43.3)	40 (66.7)	40 (69)
Daytime	1 (3.3)	3 (5)	2 (3.4)
No time	1 (3.3)	0	0
Mean years working with animals (SD)	23.6 (14.9)	26.6* (16.4)	33.8 (17)

^Denominators are shown only when missing responses and total is less than sample size.

^#^Period 1 is the 1 month prior to the disease onset or death of the case animal.

*Denominator = 57.

The participants had extensive animal care experience, ranging from an average 23.6 years (SD: 14.9) for cases to 33.8 years (SD: 17) among area controls (Table 1). Cases more frequently cared for their animals all the time (50%) compared to controls (28%), who had a higher frequency of animal care in the morning and evening (68%).

Cases had a greater frequency of being ill with anthrax or knowing another person who was ill with anthrax at any time before Period 1 than village or area controls (30% vs 14% and 7%, Table 2). Cases had 4.56 times higher odds of having an animal with anthrax when they knew someone who had anthrax or personally had a history of anthrax, compared to all controls (95% CI: 1.45, 14.30), and 7.16 times greater compared to area controls (95% CI: 1.52, 33.80) (Table 3). The comparison between cases and village controls was not statistically significant. More village controls reported knowing someone with an animal that died of anthrax, although the comparison between cases and controls was not statistically significant.

**Table 2:** Knowledge, attitudes, and practices of handlers of cattle with anthrax (‘Cases’) and handlers of matched uninfected cattle (‘Controls’) in Georgia, June 2013–May 2015.

	Cases (N = 30)	Village Controls (N = 60)	Area Controls (N = 60)
Knowledge	Count of Yes (%)	Count of Yes (%)	Count of Yes (%)
Had anthrax or know someone who had it before Period 1^#^	9/30 (30)	8/58 (13.8)	4/58 (6.9)
Had an animal die of anthrax or know someone who has before Period 1^#^	3/26 (11.5)	11/55 (20)	8/56 (14.3)
Animal species that are at risk of anthrax			
Cattle	29/30 (96.7)	54/59 (91.5)	54/60 (90)
Sheep	20/28 (71.4)	28/49 (57.1)	23/52 (44.2)
Goats	15/26 (57.7)	24/49 (49.0)	21/51 (41.2)
Pigs	9/23 (39.1)	20/49 (40.8)	12/48 (25)
Horses	13/25 (52)	22/50 (44)	15/48 (31.3)
Dogs	7/23 (30.4)	21/49 (42.9)	10/44 (22.7)
People can get anthrax from animals with anthrax	28/30 (93.3)	58/60 (96.7)	54/60 (90)
Animal anthrax can be prevented	27/30 (90)	56/60 (93.3)	45/60 (75)
Anthrax is a problem in region	21/30 (70)	37/60 (61.7)	29/60 (48.3)
Mean knowledge score (8 max) +/- *SD*	5.4 +/-1.8	5.0 +/- 2.1	4.2 +/- 2.0
**Attitudes**			
Vaccinate if free	30/30 (100)	60/60 (100)	58/60 (96.7)
Vaccinate if not free	28/30 (93.3)	58/59 (98.3)	56/59 (94.9)
**Practices**			
If an animal is ill			
Call a veterinarian	29/30 (96.7)	55/60 (91.7)	59/60 (98.3)
Treat with antibiotics	5/30 (16.7)	14/60 (23.3)	11/60 (18.3)
Slaughter	1/30 (3.3)	5/60 (8.3)	2/60 (3.3)
Separate from herd	3/30 (10)	14/60 (23.3)	8/60 (13.3)
If animal dies suddenly			
Call a veterinarian	26/30 (86.7)	48/60 (80)	50/60 (83.3)
Treat other animals with antibiotics	3/30 (10)	10/60 (16.7)	6/60 (10)
Butcher for meat	0/30 (0)	2/60 (3.3)	0/60 (0)
Sell the carcass	0/30 (0)	3/60 (5)	2/60 (3.3)
Bury the carcass	7/30 (23.3)	25/60 (41.7)	28/60 (46.7)

^#^ Period 1 is the 1 month prior to the disease onset or death of the case animal.

**Table 3:** Bivariate analyses of anthrax knowledge, attitudes, and practices among livestock handlers in Georgia.

	Cases vs All Controls	Cases vs Village Controls	Cases vs Area Controls
Variable	OR (95% CI)	OR (95% CI)	OR (95% CI)
Sex (Male Vs. Female)	4.47* (1.35, 14.83)	4.0 (1.05, 15.23)	4.41* (1.19, 16.35)
Education			
> Secondary education Vs. ≤ Secondary education	0.49 (0.19, 1.24)	0.47 (0.16, 1.35)	0.53 (0.20, 1.40)
Had anthrax/know someone who had it before Period 1^#^ (Yes Vs. No)	4.56* (1.45, 14.30)	2.99 (0.86, 10.41)	7.16* (1.52, 33.80)
Had an animal die of anthrax /know someone who had an animal die before Period 1^#^ (Yes Vs. No)	0.63 (0.15, 2.64)	0.35 (0.031, 3.36)	0.63 (0.16, 2.52)
Received anthrax information before Period 1^#^ (Yes Vs. No)	0.64 (0.22, 1.90)	0.77 (0.22, 2.68)	0.51 (0.15, 1.76)
Received anthrax information after Period 1^#^ (Yes Vs. No)	2.22 (0.76, 6.52)	1.82 (0.43, 7.79)	3.79* (1.19, 12.06)
If animal dies suddenly, bury the carcass (Yes Vs. No)	0.32* (0.12, 0.88)	0.32 (0.10, 1.05)	0.3* (0.11, 0.91)
Knowledge scale (8 max)	1.31 (1.03, 1.66)	1.18 (0.89, 1.55)	1.56* (1.14, 2.14)

Abbreviations: OR = Odds Ratio, CI = Confidence Interval.

*indicates significance at p<0.05

^#^ Period 1 is the 1 month prior to the disease onset or death of the case animal.

A comparison of receipt of information between cases, village controls, and area controls was done to verify if information was given to cases and controls within a ring vaccination area during an anthrax case investigation. Only 32% of cases reported receiving anthrax information before their animal died of anthrax (Period 1), which was similar to village controls (35%) while 45% of area controls reported receiving information. After the case animal developed anthrax (i.e., after Period 1), 81% of cases, 80% of village controls and 58% of area controls reported receiving anthrax information. The odds of a case receiving educational information on animal anthrax after Period 1 was 3.79 times the odds of area controls (95% CI: 1.19, 12.06) (Table 3).

More than 90% of all participants correctly identified cattle as susceptible to anthrax, and 55% reported sheep as susceptible. Knowledge of other animal species such as goats, pigs, horses, dogs was lower, and was lowest among the area controls (Table 3). Response rates dropped for all questions about species susceptibility after we first asked about cattle. When asked if people can get anthrax from an animal with anthrax, 93% of all responders said yes. Cases and village controls believed animal anthrax could be prevented (90%, 93% respectively); this belief was lower among area controls (75%). Cases and village controls considered anthrax a problem in their region more often than area controls (70%, 62%, and 48%, respectively).

The average knowledge score using the 8-point knowledge scale was highest among case and lowest among area controls (Table 2). With each additional point on the knowledge scale, the odds of having an animal with anthrax was 1.31 (95% CI: 1.03, 1.66) times greater when comparing cases and all controls. There was not a significant difference found between cases and either control group.

Vaccination and care practices were investigated. Willingness to vaccinate for anthrax if the vaccine was free was almost universal; willingness to vaccinate only dropped 2.7 percentage points if charged for the vaccine (Table 2). Almost all participants would call a veterinarian if their animal was sick; slightly fewer area controls would do so (Table 2). Cases more often stated they would call a government veterinarian compared to a private veterinarian (77% vs 57%). This difference was smaller among the controls, with 60% of village controls contacting a private vet and 55% contacting a government vet; the opposite breakdown was reported for the area controls. Other disease prevention actions for ill animals were infrequently reported. Village controls reported a higher frequency of disease prevention practices with sick animals, including separating it from the herd and treating with antibiotics. However, they also more frequently reported that they would slaughter the sick animal.

Practices for suddenly dead animals were similar to those for ill animals. Fewer stated they would contact a veterinarian, although it was still very common (Table 2). All three groups commonly said they would contact government vets (62–67%), and only 30–43% would call a private veterinarian, when an animal dies suddenly. Few participants stated they would butcher the carcass or sell it; the same village controls who would butcher would also sell the carcass. Fewer cases reported that they would bury the carcass compared to either control group (Table 2). The odds of burying the carcass when an animal dies suddenly is significantly lower among cases when compared to area controls. Additionally, all controls having an animal with anthrax was statistically significantly lower among those who would bury the carcass when comparing cases and all controls, and cases and area controls (Table 3).

Participants preferred educational materials in Georgian (79%), Azeri (19%), Russian (7%), and Armenian (3%); only 10 participants (6.7%) preferred materials in more than one language. The preferred source of information was from veterinarians during vaccination campaigns (64%) or when there is a health problem (18%). The second most common source was from radio and television (41%). Only 11% of respondents were interested in receiving information from leaders at village meetings, and only 5% from pamphlets.

## Discussion

Anthrax knowledge, attitudes, and practices among Georgia livestock handlers are not well known. As part of a case-control study to identify risk factors in animals, we sought to collect KAP data to guide the development of targeted interventions to reduce both animal and human anthrax. There were up to four controls for each confirmed animal anthrax death, which were matched by the animal species and by proximity to the case (within the same village and from the surrounding area outside the village). In this sub-study, we limited our evaluation to the demographics and anthrax knowledge, attitudes, and practices of case and control cattle handlers to identify factors associated with losing cattle to anthrax.

The interview process likely explains the disproportionate frequency of male cases compared to male controls; the owner/handler of the anthrax-infected animal was sought for the interview, while the control respondents were selected from the individuals at home when the investigative team arrived. Cases were less educated, had an average of 10 years less animal care experience, and cared for their animals all day. Using a shepherd to take animals to pasture during the day is common in Georgia [12], and is indicated by the owner providing animal care in the morning and evening only. From the previously mentioned study, the proportion of respondents who hired a shepherd ranged from 30–64% in the regions in which our study was conducted [12]. Together, these findings may indicate a lower socio-economic status (SES) among the cases compared to the controls, although we did not collect SES information.

Four times as many cases reported knowing a person who had anthrax or having it themselves compared to area controls. The percent of cases and village controls who knew a person with anthrax was not significantly different, as their networks likely overlapped. A greater proportion of controls reported knowing of animal anthrax deaths compared to human infections. Perhaps this experience motivated the controls to vaccinate their animals against anthrax, as a key finding from the primary case-control study found a statistically significant protective effect of anthrax vaccination within the previous two years [10].

Knowledge of anthrax among non-cattle species was lower than for cattle, although this was expected since we included only cattle handlers in our final study population. Overall anthrax knowledge was relatively low among these handlers that have decades of experience and have a somewhat high proportion of exposure to both human and animal anthrax. However, the greater level of knowledge found among the cases and village controls provides evidence that they probably received and learned from the communication messages during the case investigations and ring vaccination campaigns [13]. The percentage of cases and village controls who reported receiving information should be 100%, although recall bias, failure to provide information to all livestock handlers, or provision of materials in inaccessible formats could affect this response. In the future, the National Food Agency should consider disseminating anthrax information using evidence-based communication strategies [14, 15] to improve anthrax knowledge among livestock handlers and the private veterinarians who deliver contracted services for NFA to the livestock sector in Georgia. Additionally, information disseminated to livestock handlers outside the ring vaccination area, but located within areas known to have anthrax epizootics, could help to improve knowledge in those who also may have livestock at risk.

Despite the almost unanimous response to vaccinate regardless of whether the vaccine was free or had to be purchased, in reality animal owners were not vaccinating when the vaccine had to be purchased. A 1995 federal law instituted mandatory livestock vaccination administered by the federal government; vaccination was transitioned to livestock owners in 2007 [8]. Following this transition, both livestock and human anthrax cases increased by three- and five-fold, respectively [8].

Compared to cases, area controls less often claimed that anthrax was a problem in their region, despite at least one anthrax case occurring within 10 km of their home. As such, these same participants less often reported that anthrax could be prevented, which is consistent with lacking concern for the disease in their area.

Fewer participants were willing to contact a private veterinarian if the animal died than if the animal was ill but alive, which may indicate that participants are more likely to spend money for veterinary services if there is a chance to save an animal. Problems with veterinary services access have been reported, and only 75% of farmers in Georgia said they use veterinary services [16], which could affect the responses to requesting veterinary services. Additionally, almost all participants would contact a veterinarian if their animal was sick, but 13–20% would not contact a veterinarian if the animal died suddenly. Thus, underreporting of anthrax cases is a concern, as well as the potential environmental contamination and subsequent risk to both human and animals.

Cases were less likely to bury the animal compared to controls, which may stem from instructions not to do so, received from NFA during the anthrax case investigation. The large number of participants who report that they will bury the carcass indicates a need for training and education to prevent cutaneous anthrax and limit the environmental contamination. A recent case control study found that handlers who disposed of a livestock carcass had almost 14 times the odds of developing anthrax compared to handlers who did not dispose of a carcass [8]. Although few participants stated they would butcher, slaughter, or sell the meat or carcass in our study, another study found 88–95% of participants would consume the meat from sick or dead animals [8].

Cases and village controls did not differ significantly in their receipt of anthrax education materials after the death of the case’s animal. This indicates that veterinarians responding to the cattle anthrax death shared information with the whole village during the investigation. However, the disparity between cases and area controls indicates that these prevention activities may not be reaching beyond the anthrax-affected village. In future, veterinarians conducting anthrax investigations should extend prevention and control activities to 10–20 kilometers from the anthrax-affected village, per recommendations [1].

Limitations of the study include possible selection bias among controls. The sex disparity between cases and controls may have biased responses, given a recent study of Georgian livestock farmers that found women were reportedly more knowledgeable about an animal’s health, yet men more often make veterinary care and vaccination decisions [12]. The area controls are more likely to bury an animal that suddenly died (rather than contact their veterinarian), which could indicate that these participants have had anthrax cases that they did not report to a veterinarian. Yet, more investigation is needed since this group was also less likely to know a person with anthrax. We did not ask economic or wealth indicator questions, which may have greater impact on practices, since a previous study in Georgia postulated that meat was consumed or sold to reduce the economic loss of an animal with anthrax [8]. Additionally, the limited characteristics on which matching occurred could have led to confounding factors between cases and controls, such as the impact of herd size.

Response bias is possible, given that in addition to animal owners participating, owner’s family members and handlers/shepherds also participated and were sometimes interviewed together with the owner. Respondents may have given incorrect answers regarding the animal and its care because veterinarians were interviewing them. In addition, handlers or family members may have had different knowledge compared to the owner. Bias is likely minimal, however, given that all of the primary respondents owned the animals or were family members with the owner, and only one case reported not caring for the animal. Two shepherds participated but they were interviewed together with the owner or a family member.

The local veterinarian was frequently present during the interview because they made the introductions with the handler and the investigators, and whose presence may have influenced participants to answer practices and vaccination questions untruthfully. To ameliorate influence, the local veterinarian was briefed on his or her expected role during the interview and monitored throughout by the investigative team. Finally, recall bias about information received, and failure to differentiate correctly knowledge before and after Period 1 may artificially decrease the reporting of information received, and increase the knowledge of animal and human cases if they occurred after Period 1.

## Conclusions

The knowledge and practices gaps indicate a need for improvement in these areas. Methods aimed at addressing these gaps in Georgia may include incentives related to livestock husbandry and disease prevention (e.g., government sponsored vaccination), penalties related to food safety (e.g., fines for bypassing food safety regulations [17]), and additional educational interventions. These gaps will guide the development of educational materials in the preferred formats in Georgian, Azeri, and Russian. We initiated training of local and regional veterinarians on anthrax prevention, diagnosis, and outbreak response in the region surrounding an animal anthrax illness or death. Additionally, veterinarians were provided materials from which to educate livestock farmers about anthrax during vaccination campaigns and when providing routine veterinary care.

## Supporting information

S1 Data (XLSX)

S1 Questionnaire (DOCX)

S2 Questionnaire (DOCX)
